# The Etiology of Cancer

**Published:** 1898-06

**Authors:** Wm. B. Clarke

**Affiliations:** Indianapolis, Ind.


### THE ETIOLOGY OF CANCER.

Wm. B. Clarke, M. D., Indianapolis, Ind.

In a paper entitled "The Treatment of Cancer," by Dr. Wm. B. Clarke, of Indianapolis, Ind., read by him in person, by invitation, before the Kentucky Homœopathic Medical Society at Frankfort, May 25th, the question of the cause of the disease carcinoma is interestingly handled, as follows:

The malignancy and fatality of carcinoma and the long continued Indian-like torture of its victims characteristic of the disease justifies the popular horror of its very name, and its appalling frequency and rapid increase of late years may well challenge the closest attention of the medical profession of the civilized world. I say civilized world, for in an editorial in its June, 1897, issue the *Philadelphia Medical Summary* says: "Cancer is a disease of civilization, and was not much known until a few generations ago. So far there is no known remedy for this calamitous affection, . . . and scarcely an alleviation possible of its sufferings; . . . it remains among the unsolved and perplexing riddles of science, and promises no indication of less severity." I also say civilized world because Jeancon, in his *Diseases of the Sexual Organs*, says, "Cancer is unknown in Africa"—a reference I shall allude to later. But with us it steadily pursues its relentless march, slaying the Grants and Gladstones of the world with as little leniency as it displays toward their humblest and most helpless followers.

Now a paragraph as to its frequency and increase, as spoken of in opening: In 1895 Dr. Joseph D. Bryant, of New York, before the New York Medical Association, presented statistics showing the deaths from cancer in the United States to have been 9 per 100,000 living in 1850; 11.7 in 1860; 16 in 1870; [26 in 1880—U. S. Census], and 35.5 in 1890, nearly quadrupled in forty years. The New York State

Board of Health report shows 3,454 deaths in that State in 1895, or nearly twice as many as ten years before. And it is worse abroad, the ratio of increase being much the same but the figures being larger—*i. e.*, 67.5 in England in 1890, and 52.8 in Prussia (Dennis's *Surgery*, Vol. IV, p. 92). As to the number of deaths in the United States the same work, p. 91, quotes Dr. J. S. Billings, U. S. Army, the noted statistician, as authority for 18,536 for the year ending May 31st, 1890; "much below the true number," as it includes only absolutely officially reported cases, and including no sarcomas, "or more than the sum total of deaths due to erysipelas, tetanus, hydrophobia, lightning, typhilitis, gun-shot wounds, joint diseases, together with other well known surgical affections" (Dennis's *Surgery*, Vol. IV, p. 91). Now it will not do to advance the specious argument, better diagnoses now than then, for by its very presentation the argument fails, as sarcomas and "any old thing" in the way of tumor is now excluded, while then often included.

The general subject of cancer is far too large to be even thinly veneered in a single essay, but it is appropriate on an occasion like the present to devote a little attention to the vitally interesting question of the etiology of the disease, about which all the book authorities, from Billroth and Virchow down, agree in saying there is almost nothing positively known. They tell us that age, irritation, heredity, sex, menopause, vegetable growths and injury are predisposing factors, but leave us to burst in ignorance as to the actual causes. This disease is the grand type of the class called heterologous hyperplasia, being characterized by a prodigiously abnormal proliferation of epithelium.

Malignant growths are at first purely local and benign, carcinoma originating from cells which belong to the epiblastic or hypoblastic structures, epithelial, while sarcomas are from the mesoblastic, connective tissue. Now, it takes twenty-one years to make a man, and but four to make a cow.

As cancer is a disease characterized by the rapid imposition of cells, is it safe to put the rapid growing cells or protoplasm into the slow-growing cells, as is done in vaccination and the various forms of blood assassination now rife in civilized countries, often, indeed, enforced by law or health(?) board regulation? Granted that the practice does not cause the disease outright, as it does consumption, is it not at least reasonable to suppose that it predisposes us toward it? And we are now reaping the harvest of the seed so generally introduced half a century ago—Prussia's vaccination law having been passed in 1835, and England's in 1853. And the figures are larger abroad, as quoted above, because the practice of vaccination was more generally introduced some years earlier there. And consumption has steadily increased from the time when young Phipps, Jenner's first vaccination victim, and also his own frequently-experimented-on son, died of it. Much authoritative testimony could here be cited did time and space allow. I will only say that our census shows nearly one-half of the deaths from cancer in proportion to population in 1880 to have been among the colored people, and this in face of the fact, previously alluded to, that "cancer is unknown in Africa." That their proportionate death-rate from cancer here does not equal or even exceed that of the whites may be explained by the fact that they are more prejudiced against vaccination, and so more of them escape the esoteric rite and its dangers. New York vaccinates freely and by force, and its cancer deaths double in ten years.

But let the causation of diseases by vaccination and the tendency to cause them be studied carefully, and the mystery which now puzzles us with regard to cancer may be largely cleared away. It is high time to call an abrupt halt in the world-wide prevalence of the practice of these various "scientific" assassinations of the blood of our domestic animals, especially cows, horses, and dogs. It is not only degrading and debasing them, now and for their posterity, but is sure to boomerang disastrously upon the physical welfare of the human race. Who can estimate the havoc even now wrought along this line? And why should we longer countenance this recrudescence of superstition, or longer worship this fetich of hygiene, this useless and dangerous procedure?

